# Supplementary material for: Performing highly parallelized and reproducible GWAS analysis on biobank-scale data
Source: NAR Genom Bioinform. 2024 Feb 7;6(1):lqae015. doi: 10.1093/nargab/lqae015 (PMC10849172; doi:10.1093/nargab/lqae015)
Supplement: lqae015_Supplemental_File [file lqae015_supplemental_file.pdf]

Supplemental Material

Performing highly parallelized and reproducible GWAS analysis on biobank-scale data

Sebastian Schönherr[1*], Johanna Schachtl-Riess[1*], Silvia Di Maio[1], Michele Filosi[2], Marvin Mark[1], Claudia Lamina[1], Christian Fuchsberger[1,2], Florian Kronenberg[1], Lukas Forer[1]

[1] Institute of Genetic Epidemiology, Medical University of Innsbruck, Innsbruck, Austria

[2] Institute for Biomedicine, Eurac Research, Affiliated Institute of the University of Lübeck, Bolzano, Italy

* contributed equally

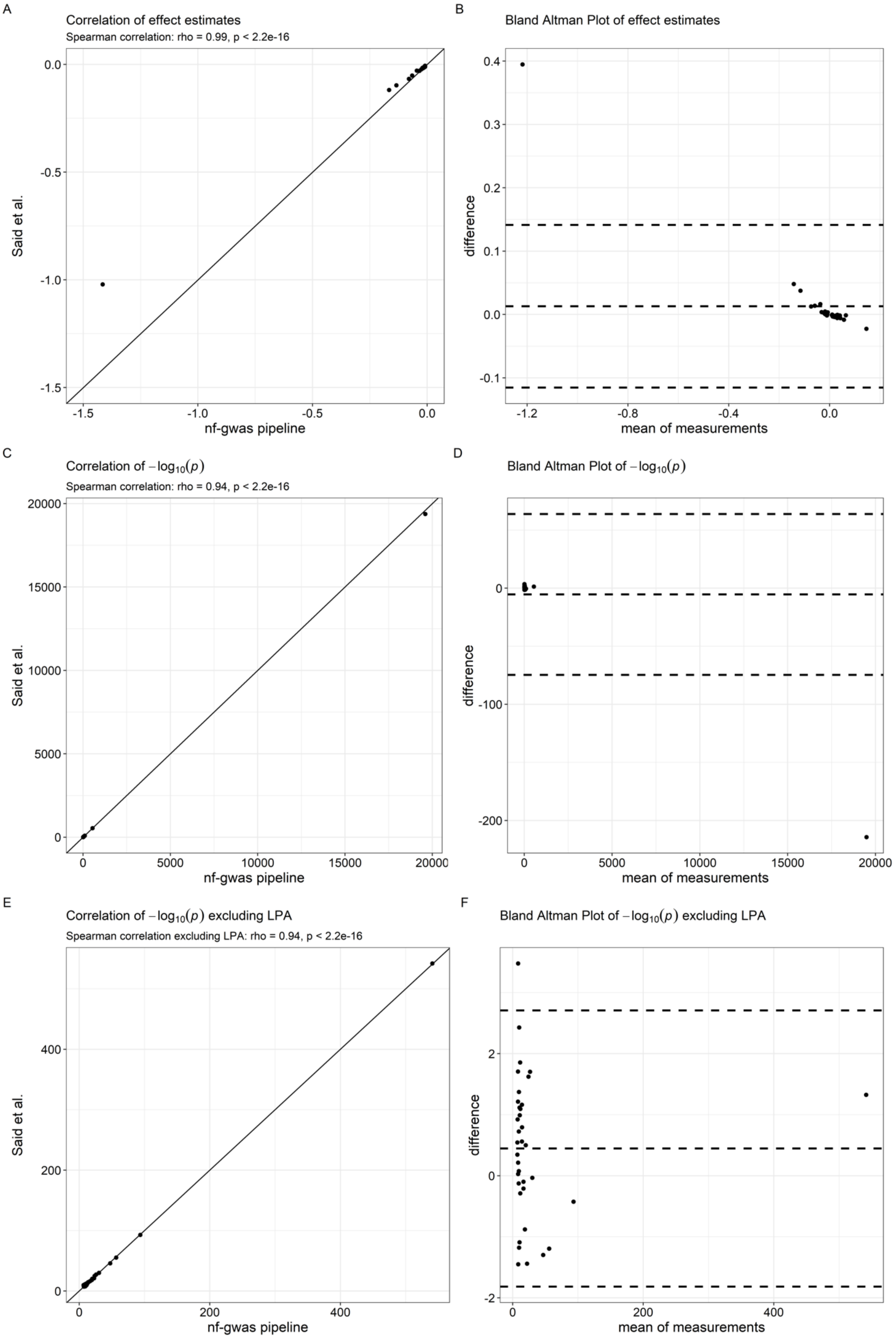
A
Correlation of effect estimates
Spearman correlation: rho = 0.99, p < 2.2e-16
Said et al.
nf-gwas pipeline
0.0
-0.5
-1.0
-1.5
B
Bland Altman Plot of effect estimates
difference
mean of measurements
0.4
0.3
0.2
0.1
0.0
-0.1
-1.2
-0.8
-0.4
C
Correlation of $-\log_{10}(p)$
Spearman correlation: rho = 0.94, p < 2.2e-16
Said et al.
nf-gwas pipeline
20000
15000
10000
5000
0
D
Bland Altman Plot of $-\log_{10}(p)$
difference
mean of measurements
-100
-200
E
Correlation of $-\log_{10}(p)$ excluding LPA
Spearman correlation excluding LPA: rho = 0.94, p < 2.2e-16
Said et al.
nf-gwas pipeline
400
200
F
Bland Altman Plot of $-\log_{10}(p)$ excluding LPA
difference
mean of measurements
2
-2

Figure S1: Comparison of sentinel SNPs of the Lp(a) GWAS by Said et al. to our pipeline. *Plots A-D contain 37 variants (rs139970591 and rs531017143 were not available in our GWAS results) and plots E and F additionally exclude rs10455872 (LPA). A, C and E depict correlation plots and the black line denotes equal values between both studies. B, D and F depict Bland Altman plots depict mean and difference with x = Said et al. and y = nf-gwas pipeline of effect estimates on RINTed Lp(a) in B and* $-\log_{10}(p)$ *in D and F and the dashed lines represent mean ± 2 SDs of differences (95% limits of agreement).*

Table S1: *Comparison of effect estimates and -$log_{10}(p)$ for tophits of the GWAS on RINTed Lp(a) between our pipeline and Said and colleagues. Chr: Chromosome; LOG10P : -$log_{10}(p)$*

			nf-gwas							Said et al.	
rsID	Chr	Gene	A0	A1	A1FREQ	INFO	BETA	SE	LOG10P	BETA	LOG10P
rs182050989	1	NUDC	T	C	0.97	0.99	0.03	0.01	8.67	0.03	11.10
rs11591147	1	PCSK9	T	G	0.98	1.00	0.05	0.01	11.21	0.04	12.30
rs599839	1	CELSR2, PSRC1	A	G	0.24	1.00	-0.02	0.00	23.38	-0.02	25.00
rs934197	2	APOB	A	G	0.67	1.00	-0.02	0.00	19.80	-0.01	20.30
rs1047891	2	CPS1	A	C	0.68	1.00	0.02	0.00	30.13	0.02	30.10
rs115562858	3	IP6K2, PRKAR2A	C	G	0.98	0.98	0.04	0.01	8.27	0.04	8.30
rs77601270	3	RBM15B, VPRBP	C	G	0.98	0.98	0.04	0.01	9.45	0.04	9.52
rs645040	3	MSL2	T	G	0.23	1.00	0.02	0.00	22.74	0.02	21.30
rs2045592	4	AFF1	G	C	0.62	1.00	-0.01	0.00	8.18	-0.01	8.40
rs1908961	4	ADH7	G	A	0.79	0.99	-0.01	0.00	10.54	-0.01	12.40
rs114816312	4	PLA2G12A	T	C	0.99	1.00	0.07	0.01	9.88	0.06	11.00
rs143500414	6	TULP4	C	T	0.98	0.87	-0.07	0.01	10.31	-0.05	11.30
rs141117870	6	FNDC1	T	C	0.98	0.96	-0.17	0.01	93.43	-0.12	93.00
rs10455872	6	LPA	G	A	0.93	1.00	-1.42	0.00	19593.70	-1.02	19379.40
rs191369471	6	PARK2	G	C	0.99	0.85	-0.13	0.02	16.61	-0.10	16.40
rs34115226	7	NFE2L3	CG	C	0.20	0.99	0.01	0.00	6.52	0.01	10.00
rs35797675	7	BAZ1B	G	T	0.79	0.98	0.01	0.00	8.97	0.01	9.70
rs4841132	8	PPP1R3B	G	A	0.09	1.00	-0.02	0.00	11.09	-0.02	10.00
rs17411113	8	LPL	G	C	0.90	1.00	-0.03	0.00	19.18	-0.02	18.30
rs61887548	11	CHKA	C	T	0.98	1.00	0.03	0.01	8.85	0.03	10.22
rs2657878	12	MIP, SPRYD4, GLS2	T	C	0.82	0.99	0.01	0.00	9.15	0.01	7.70
rs139097404	15	STRC, CATSPER2	C	T	0.98	0.97	0.06	0.01	25.70	0.05	27.40
rs1532085	15	ALDH1A2	G	A	0.39	1.00	0.01	0.00	7.34	0.01	9.05
rs247616	16	CETP	T	C	0.68	1.00	0.03	0.00	47.30	0.02	46.00
rs1110572	16	PRMT7, SMPD3	G	A	0.88	1.00	-0.02	0.00	11.81	-0.02	11.52
rs34042070	16	TXNL4B, HP, HPR	G	C	0.81	0.99	-0.02	0.00	13.54	-0.02	14.70
rs139970591	17	PEMT	NA	NA	NA	NA	NA	NA	NA	0.01	12.00
rs3785549	17	PSMD3	C	T	0.46	0.99	-0.01	0.00	7.08	-0.01	8.00
rs1801689	17	APOH	C	A	0.97	1.00	-0.08	0.01	56.49	-0.07	55.30
rs77542162	17	ABCA6	G	A	0.98	1.00	-0.05	0.01	13.96	-0.03	14.52
rs2292642	17	PGS1	T	C	0.40	1.00	-0.01	0.00	6.98	-0.01	7.52
rs874492	19	CLEC4M	T	A	0.68	0.99	-0.01	0.00	7.05	-0.01	7.40
rs138294113	19	LDLR	T	C	0.88	1.00	0.02	0.00	16.62	0.02	16.52
rs1065853	19	TOMM40, APOE, APOC1	T	G	0.92	1.00	0.16	0.00	540.67	0.13	542.00
rs35866622	19	FUT2, MAMSTR, RASIP1	T	C	0.52	0.99	-0.01	0.00	13.91	-0.01	14.70
rs8736	19	TMC4, MBOAT7	T	C	0.56	0.99	-0.01	0.00	10.33	-0.01	9.15
rs73075609	20	GPCPD1	T	C	0.97	0.98	-0.03	0.01	9.28	-0.03	9.15
rs531017143	20	ZGPAT, LIME1, SLC2A4RG	NA	NA	NA	NA	NA	NA	NA	-0.01	10.40

Table S2: *Comparison of features of existing pipelines.*

GitHub	**nf-gwas**<br>https://github.com/genepi/nf-gwas	**nf-gwas-pipeline**<br>https://github.com/montilab/nf-gwas-pipeline	**H3AGWAS**<br>https://github.com/h3abionet/h3agwas	**BIGwas**<br>https://github.com/ikmb/gwas-assoc
**Association software**	REGENIE	GENESIS, GMMAT	gcta, plink, gemma, Bolt-LMM, REGENIE, saige, fast-lmm	PLINK, SAIGE
**Input formats**	VCF, BGEN	VCF or gds	depends on software	VCF
**Quantitative traits**	yes	yes	yes	yes
**Binary traits**	yes	yes	yes	yes
**>1 phenotype**	yes	no	yes	no
**Step 1 QC**	yes	not applicable	not applicable/no	Not applicable
**Single-variant testing**	yes	yes	yes	yes
**Rare-variant/gene-based testing**	yes	yes	no	no
**GxE testing**	yes	no	yes	no
**GxG testing**	yes	no	no	no
**Conditional analysis**	yes	no	no	no
**longitudinal analysis**	no	yes	no	no
**Report**	yes	yes	yes	yes
**Interactive report**	yes	no	no	no
**Tabix-indexed (LocusZoom ready)**	yes	no	no	no
**Annotations**	nearest gene + rsID	ANNOVAR	In separate pipeline	no
**Lift over**	yes	no	no	In separate QC pipeline

**option**				
**PC generation**	no	yes	no	yes
**Addition of own PCs**	yes	yes	yes	no
**Software tests**	yes (nf-test)	no	no	no
